# Dental Rehabilitation After Microvascular Reconstruction of Segmental Jaw Defects: A Ten-Year Follow-Up

**DOI:** 10.3390/jcm14020628

**Published:** 2025-01-19

**Authors:** Katharina Zeman-Kuhnert, Alexander J. Gaggl, Gian B. Bottini, Benjamin Walch, Christoph Steiner, Georg Zimmermann, Christian Brandtner

**Affiliations:** 1 Department of Oral and Maxillofacial Surgery, University Hospital of Salzburg, Paracelsus Medical University, 5020 Salzburg, Austria; a.gaggl@salk.at (A.J.G.); g.bottini@salk.at (G.B.B.); b.walch@salk.at (B.W.); c.steiner@salk.at (C.S.); c.brandtner@salk.at (C.B.); 2 Team Biostatistics and Big Medical Data, IDA Lab Salzburg, Paracelsus Medical University, 5020 Salzburg, Austria; georg.zimmermann@plus.ac.at; 3 Research Programme Biomedical Data Science, Paracelsus Medical University, 5020 Salzburg, Austria

**Keywords:** oral cancer, dental rehabilitation, microvascular jaw reconstruction, reconstructive surgery, osteonecrosis, cleft lip and palate

## Abstract

**Background/Objectives**: Defects in maxillary and mandibular continuity are common in maxillofacial practice. They can occur after trauma, osteonecrosis, congenital jaw deformities, or surgical resection of benign or malignant tumours. Reconstruction with microvascular bone flaps and subsequent prosthetic rehabilitation is considered the contemporary first line treatment. This study assessed the extent to which the underlying disease influences the treatment course of microvascular segmental jaw reconstruction. **Methods**: A retrospective review of prospectively collected data from all patients who underwent microvascular segmental jaw reconstruction from January 2011 to December 2020 was completed. The course of treatment as well as the process of dental rehabilitation was assessed. **Results**: Two hundred patients were included in the study. A total of 15.5% of patients were fitted with a conventional removable prosthesis, and implant-supported prosthetic restoration could be realized in 53.5% of patients. However, dental rehabilitation was not possible in 31.0% of patients. The outcomes of prosthetic restoration showed a statistically significant difference between the different underlying diseases (*p* < 0.0001). About 50% of patients with malignant diseases and osteonecrosis remained without dental rehabilitation. In contrast, more than 90% of patients with jaw continuity defects, due to benign diseases or malformations, were able to receive an implant-supported prosthesis. Among the diagnostic groups, there was a significant difference regarding the number of implants placed (*p* < 0.0001). There was a significant correlation between increased incidence of complications and the size of the bone flaps. **Conclusions**: The underlying disease influenced significantly the treatment course and the outcome of dental rehabilitation after microvascular jaw reconstruction.

## 1. Introduction

Defects in maxillary and mandibular continuity are common in maxillofacial practice, arising from various causes including trauma, infection, osteonecrosis, congenital jaw deformities, or surgical resection of tumours. These defects can lead to facial disharmony, impaired speech, difficulties in mastication, and dietary restrictions, significantly affecting patients’ quality of life [1,2,3,4]. The primary goal of current microvascular reconstruction techniques is to restore the structural integrity of the jaw, replace damaged soft tissue, and provide a scaffold for dental rehabilitation. However, beyond restoring the bony jaw continuity, functional dental rehabilitation is key to achieving positive long-term outcomes. Without dental rehabilitation, patients may experience compromised mastication, reduced oral competence, and impaired speech intelligibility [4,5].

Dental rehabilitation offers the potential to restore efficient mastication, improve swallowing, and enhance speech function, contributing to improved aesthetic outcomes and overall quality of life [6,7,8]. Yet, the process is complex and must be individualized, as both the anatomical conditions of the reconstructed jaw and the underlying disease must be considered. Insufficient bone height or inadequate soft tissue coverage can hinder the success of tissue-borne prostheses, while the lack of adequate dentition limits options for anchoring removable dentures [9].

Additionally, the nature of the underlying disease plays a crucial role in shaping the approach to jaw reconstruction and dental rehabilitation. In malignant conditions, large defects often result from necessary surgical resections, and prosthetic rehabilitation can be further complicated by the side effects of medications or irradiation. In these cases, conventional removable dentures may not be suitable due to the inability of the denture-bearing mucosa to tolerate mechanical loading [10]. Implant-supported prostheses have been shown to offer superior dental rehabilitation [4,11,12], yet these may not be feasible for all individuals, leaving some patients edentulous and reliant on a soft diet. In contrast, patients with benign diseases typically present with smaller defects, do not require adjuvant therapies, and tend to be in better overall health, which may facilitate the rehabilitation process.

This study aims to evaluate the outcomes of dental rehabilitation in patients who underwent microvascular reconstruction of segmental jaw defects at a single centre over a 10-year period, regardless of the underlying disease. Specifically, the study seeks to determine how the nature of the underlying disease influences the healing process and the success of dental rehabilitation.

## 2. Materials and Methods

### 2.1. Patients

A retrospective review of prospectively collected data from all patients who underwent microvascular reconstruction of segmental jaw defects with a bone flap at the Department of Oral and Maxillofacial Surgery at the University Hospital Salzburg, Austria, between January 2011 and December 2018 was completed. All patients were followed up from the day of reconstruction until the end of 2020, allowing a minimum follow-up time of 2 years.

### 2.2. Variables Reviewed

The course and outcome of dental rehabilitation, as well occurrence of complications requiring surgical intervention during this 10-year period were recorded. For dental implants placed in the microvascular flap, the implant loss and survival rates were analysed.

In addition, the medical records were reviewed to record patient’s demographics, indications for jaw reconstruction, affected jaw, type of microvascular flap, and length of the reconstruction.

### 2.3. Statistical Analysis

Initially, associations between variables were descriptively analysed using Spearman’s correlations or counts and percentages, as appropriate. In a second exploratory step, Fisher’s exact tests and generalizations were conducted to examine associations between categorical variables. These tests were chosen due to very small cell counts for some factor level combinations. For comparing transplant sizes between groups, the Wilcoxon–Mann–Whitney test and a Kruskal–Wallis-type test were used [13]. The corresponding effect measure, which is the relative effect, has been reported, too. The Relative Effect (Rel. Eff.) is the probability that outcome values in one group tend to be higher than the values in another group (e.g., when comparing subjects experiencing a certain complication with subjects without that complication). The two-sided significance level was set to alpha = 0.05 for all statistical tests. Statistical analyses were conducted using the statistical software package R version 4.0.2 [14].

## 3. Results

A total of 207 patients underwent segmental jaw reconstruction at the University Hospital Salzburg between January 2011 and December 2018. Seven patients were excluded because of loss to follow-up, leaving two hundred patients for analysis. Among them, 86 were female (43.0%), and the mean age was 54.8 years (range, 15–89 years).

The indications for microvascular jaw reconstruction included malignant tumours (56.5%, *n* = 113), benign tumours/cysts (5.0%, *n* = 10), malformations (12.0%, *n* = 24), osteonecrosis (6.0%, *n* = 12), and other benign diseases, like trauma and gunshot sequelae, Noma, osteomyelitis, alveolar ridge atrophy, and non-union (20.5%, *n* = 41).

The mandible was reconstructed in 123 patients, while the remaining 77 underwent maxillary reconstruction (Table 1).

The average length of the reconstructed jaw for the whole cohort was 6.5 cm (range, 1.0–15.0 cm). The average length for malignancies and osteonecrosis was 7.6 cm (range, 3.0–13.5 cm) and 6.6 cm (range, 5.0–10.0 cm), respectively, and for benign tumours and others 5.1 cm (range, 3.0–13.5 cm) and 5.4 cm (range, 2.5–11.5 cm), while for malformations 3.5 cm (range, 2.0–10.0 cm).

The microvascular graft was selected according to an internal departmental scheme depending on the size and morphology of the bone defect and the affected jaw (Table 2) [15].

### 3.1. Dental Rehabilitation

After microvascular jaw reconstruction, 31 (15.5%) patients received a conventional removable prosthesis, with 16 (8.0%) receiving complete dentures and 15 (7.5%) receiving partial dentures. Implant-supported prosthetic restoration was achieved in 107 (53.5%) patients, which was fixed in 46 (23.0%) patients and removable (bar-retained or locator-retained) in 61 (30.5%) patients. Dental rehabilitation was not possible in 62 (31.0%) patients.

The outcomes of prosthetic restoration for the cohort showed a significant difference between the different underlying diseases (*p* < 0.0001) and between maxilla and mandible (*p* = 0.0003) (Table 3).

We observed no statistically significant association between prosthetic outcome and gender (*p* = 0.8551).

A significant association was found between bone flap length and prosthetic outcome (*p* < 0.0001). If the reconstructed jaw was longer, it was more frequently restored with conventional full dentures (Rel. Eff. = 0.60) or no prosthetic restoration at all (Rel. Eff. = 0.62).

Shorter reconstructed jaw defects were more likely to be restored with implant-supported fixed dentures (Rel. Eff. = 0.25).

### 3.2. Dental Implants

Implants (Institute Straumann AG, Basel, Switzerland) were inserted using templates after precise implant position planning based on 3D image data. A total of 112 (56.0%) patients underwent at least one implant placement. A total of 415 implants were placed with a median of four implants per patient (range, 1–8 implants).

A total of 28 (25.0%) of these 112 patients had at least one implant loss. Overall, 53 (12.8%) out of 415 implants were lost; the median was one implant per patient (range, 1–5 implants). Implant survival in the population was 87.2%.

The distribution of implant insertions and losses within the different diagnoses can be seen in Table 4. Among the diagnostic groups, there was a significant difference regarding implant placements (*p* < 0.0001), but no difference regarding implant losses (*p* = 0.0906).

A total of 63 (81.8%) of the 77 patients with maxillary reconstruction received at least one implant, whereas only 49 (39.8%) of the 123 patients with mandibular reconstruction received at least one implant. The difference was statistically significant (*p* < 0.0001).

A total of 19.0% (*n* = 12) lost at least one implant in the maxilla and 32.7% (*n* = 16) in the mandible. The difference was not statistically significant (*p* = 0.1249).

Gender had no effect on the number of implants placed (*p* = 0.303) or implant losses (*p* = 0.223). There was no correlation between age and implant loss (r = 0.11).

There was a statistically significant association between smaller bone flaps and implant insertion (Rel. Eff. = 0.32; *p* < 0.0001). The amount of implant loss was not significantly influenced by graft size (Rel. Eff. = 0.59; *p* = 0.2903). Significantly fewer implants were placed in the presence of a soft tissue flap (*p* < 0.0001), but it had no effect on implant losses (*p* = 1.00).

### 3.3. Complications and Reoperations

Soft tissue corrections were performed in 89 patients (44.5%); these were mainly planned pre-prosthetic procedures (e.g., vestibuloplasty) or aesthetic corrections (e.g., to the lips).

A total of 66 patients (33.0%) had at least one complication that required surgical treatment. Complications with the osteosynthesis material (infected or uncovered) occurred in 29 (14.5%) patients and with the microvascular bone graft occurred in 13 (6.5%) patients. A total of 12 (6.0%) patients lost their bone flap. 14 (7.0%) patients received a new or additional microvascular bone flap. Complications with the soft tissue paddle (thrombosis or haemorrhage) occurred in 8 patients (4.0%), and there was no soft tissue loss.

A total of 13 patients (6.5%) had other complications at the reconstruction site (e.g., fistula, pseudarthrosis, and wound dehiscence). Four patients (2.0%) were reoperated on due to other complications near the reconstruction site (osteonecrosis and osteomyelitis).

Reoperation due to tumour recurrence at the site of origin had to be performed in 10 of the 113 cancer patients (8.8%) and in 3 of the 12 patients with osteoradionecrosis (25.0%). Due to recurrence/second tumour outside the area of microvascular reconstruction, 6 of the 113 cancer patients (5.3%) had to be reoperated on.

More detailed information on complications and reoperations can be found in Table 5.

Reoperations due to the following causes “complications with osteosynthesis material”, “soft tissue corrections”, “bone flap loss”, “tumour recurrence (site of origin)”, and “new/additional bone flap” showed significant differences between the individual diagnoses (*p* < 0.05). Significant differences were found between maxilla and mandible for “complications with osteosynthesis material”, “soft tissue corrections”, and “other complication (site of reconstruction)” (*p* < 0.05). No differences were attributable to gender (*p* > 0.05).

Further, the presence of a soft tissue paddle did not lead to a higher incidence of reoperations than bone grafts without a soft tissue paddle (*p* > 0.05).

There was a significant correlation between increasing bone grafts and incidence of “complications with the osteosynthesis material”, “other complication (site of reconstruction)”, and “other complication (next to reconstruction area)”. Smaller flaps were significantly more likely to have “soft-tissue corrections” (Table 6).

## 4. Discussion

In this study, the authors followed up patients who underwent reconstruction of segmental jaw defects with a microvascular bone flap over a 10-year period. Particular attention was paid to the course and success of dental rehabilitation, as this is essential for the overall rehabilitation of orofacial functions such as chewing, swallowing, and speaking, as well as general well-being [4,5,7].

Since individual challenges arose, the aim of the study was to find out to what extent the underlying disease influenced the course of treatment and the success of microvascular jaw reconstruction and dental rehabilitation.

### 4.1. Dental Rehabilitation

Functional dental rehabilitation was achieved in 69.0% of the study population, and more than 50% of patients had an implant-supported dental rehabilitation. This result is satisfactory, as dental implantology is the most effective method for the adequate restoration of chewing function and dental aesthetics, as conventional dentistry is often unsuitable due to the altered anatomy, the vestibular space, and the retention capacity of the reconstructed jaw [12,16].

The underlying diseases significantly influenced the results of dental rehabilitation. In about half of the patients with malignant diseases and half of the patients with osteonecrosis, a prosthetic restoration was not possible. These results are similar to the existing literature, in which functional dental rehabilitation was achieved in only 31.4% to 42.9% of reconstructed oral cancer patients [2,17,18]. The effects of cancer treatment of the oral cavity have far-reaching consequences in terms of a functional dental rehabilitation. Despite advanced reconstructive surgical techniques, these cannot sufficiently restore the sensory–motor functions lost through tumour resection and often fail to provide adequate support for conventional tissue-borne prostheses [19]. In addition, in cases of malignant tumours, fibrosis and xerostomia after radiotherapy further reduce the possibility to use conventional removable prostheses. The irradiated mucosa is often unable to tolerate the friction generated by tissue-supported prostheses. Therefore, the literature recommends the use of osseointegrated implants that are inserted into the microvascular bone flap to ensure a stable prosthetic fit [11,12,20]. However, implants must be used with caution in both oral cancer and osteonecrosis patients and strict criteria must be applied to the selection of patients.

In contrast, more than 90% of patients with benign tumours, malformations, and other benign diseases were able to receive an implant-supported prosthesis.

There was a statistically significant difference between the maxilla and mandible in relation to the outcome of dental rehabilitation: While around 80% of patients who underwent maxillary reconstruction received implant-supported dentures, there was a relatively high percentage of patients (42.3%) for whom no dental rehabilitation at all was possible in the mandible. This can be explained by the fact that the mandible was affected in 2/3 of all malignancies and in over 90% of all osteonecrosis patients. The greater success of implant-supported restorations in the maxilla was due to the fact that almost 90% of patients with malformations, such as cleft lip and palate, received a maxillary reconstruction. In the malformation group, all but one patient had an implant-supported prosthesis.

Surprisingly, there was an association between bone flap size and prosthetic outcome: the smaller the flap, the higher the likelihood of an implant-supported fixed prosthesis. It was shown, however, that the malformation group had the smallest bone flaps with an average length of 3.5 cm, while the malignancies and osteonecroseis had the longest reconstructions at 7.6 cm and 6.6 cm, respectively.

### 4.2. Dental Implants

At least one dental implant was placed in 112 patients (56.0%). Of these, 107 patients received an implant-based dental rehabilitation, which is encouraging. In contrast, Ostrander et al. reported a lower rate of dental implant placement (20.8%), with only 6.3% receiving a final prosthesis after free flap reconstruction over 5 years in their single-centre study [21]. However, in contrast to the present study, their patients had a much higher incidence of malignant diseases and did not receive dental implants in irradiated bone, which may explain the different results.

Implant survival in our population was 87.2%, which is similar to the literature. In a systematic review and metanalysis of implants survival rates in vascularized bone free flaps, six studies provided an overall 5-year implant survival of 85.4% [9,22,23,24,25,26].

When considering the implant insertions in relation to the underlying diseases, there were significant differences. All patients with benign tumours and all but one of the patients with malformations received implants, while only 30% of patients with malignancies and 50% of patients with osteonecrosis received implants. In the case of benign diseases and malformations, good oral hygiene, few concomitant diseases, young age, and healthy bone next to the reconstruction site favour implant placement. Patients with malignant diseases, on the other hand, present often with oral cavity carcinoma risk factors such as smoking and alcoholism, as well as poor oral hygiene, which can be contraindications for implantation. In addition, many oral cancer patients are in need of adjuvant radiotherapy. Radiation exposure of the reconstructed jawbone has a negative impact on implant survival rates, due to radiotherapy-reduced vascularization and tissue fibrosis, as well as lowering infection resistance, which could lead to osteoradionecrosis. Studies have already shown that dental implant survival rates were significantly higher in bone flaps without radiotherapy exposure than in irradiated bone flaps [9,20,27]. Dental implant survival rates in irradiated bone flaps can increase with implant placement before irradiation or delaying implantation 6–12 months after radiotherapy. However, an implantation should still be decided individually based on health status including comorbidites, and the patient’s motivation and compliance.

The location of jaw reconstruction (maxilla or mandible) had no significant impact on implant loss, which is consistent with the literature [22].

### 4.3. Complications/Reoperations

The most common planned surgical interventions after microvascular jaw reconstruction were soft tissue corrections, which were mainly pre-prosthetic procedures such as vestibuloplasties or soft tissue corrections. These were performed significantly more frequently in malformations and benign diseases, as well as in the maxilla. There was also a correlation with smaller flap sizes. As previously noted, the malformations had the smallest graft size (3.6 cm) and 90% of the reconstructions were in the maxilla. In addition, more than 95% of the patients with benign diseases and malformations received at least one implant.

The overall postoperative complication rate was 33%, which is consistent with results of previous studies (27–48%) [28,29]. The most common complications were due to the osteosynthesis material, representing 14.5% of the complications in the overall group.

The osteonecrosis group demonstrated the highest complication rate with the osteosynthesis material (42%). Complications with the osteosynthesis material also occurred more frequently in the mandible, similar to previous studies where plate removal after mandibular reconstruction was due to postoperative complications, such as fistulae (12.2%), and plate exposure or infections (16%) [30,31].

The total bone flap success rate was 94.0%, which is consistent with other reports (91.7–98.7%) [17,29,32]. The osteonecrosis group had the highest flap loss rate at 25%. The above flap loss rate could be due to the severe compromise of the vascularity in the recipient bed, in the flap itself, and to the compromised healing capacity of the patient as a side effect of antiresorptive medications [33,34].

One limitation of the study is that the status of the residual dentition was not taken into account, as the residual dentition does have an influence on the patient’s desire for dental rehabilitation. In some cases, dental rehabilitation of the reconstructed jaws was not performed because the patient was satisfied with the residual dentition or accepted postoperative dietary restrictions and did not want to incur the additional costs and appointments involved in a complex dental rehabilitation.

## 5. Conclusions

After microvascular reconstruction of segmental jaw defects, functional dental rehabilitation was achieved in 70% of all patients, with 50% receiving an implant-supported denture. Benign underlying diseases and malformations received implant-supported dentures in over 95% of cases, while almost 50% of osteonecrosis and malignant diseases received no dental rehabilitation.

## Figures and Tables

**Table 1:** Reconstructed jaw within the underlying diseases.

	Malignancy (*n* = 113)	Benign Tumour/Cysts (*n* = 10)	Malformation (*n* = 24)	Osteonecrosis (*n* = 12)	Others (*n* = 41)
Maxilla	27 (23.9%)	5 (50.0%)	21 (87.5%)	1 (8.3%)	23 (56.1%)
Mandible	86 (76.1%)	5 (50.0%)	3 (12.5%)	11 (91.7%)	18 (43.9%)

**Table 2:** Flap related information.

Variable	Specification	No. of Patients	Percentage
Microvascular flap	Iliac crest	77	38.5
	Fibular	55	27.5
	Scapula	11	5.5
	Femur	54	27.0
	Combination (iliac crest + femur)	1	0.5
	Other (radius, humerus)	2	1.0
Osteocutaneous flap	Yes	57	28.5
	No	143	71.5

**Table 3:** Outcome of dental rehabilitation.

		No Denture	Complete Denture	Partial Denture	Implant-Supported Removable Denture	Implant-Supported Fixed Denture
Underlying disease	Malignancy	55 (48.7%)	16 (14.2%)	11 (9.7%)	25 (22.1%)	6 (5.3%)
	Benign tumour	0	0	0	1 (10.0%)	9 (90.0%)
	Malformation	0	0	1 (4.2%)	11 (45.8%)	12 (50.0%)
	Osteonecrosis	6 (50.0%)	0	1 (8.3%)	5 (41.7%)	0
	Others	1 (2.4%)	0	2 (4.9%)	19 (46.3%)	19 (46.3%)
Reconstructed jaw	Maxilla	10 (13.0%)	5 (6.5%)	2 (2.6%)	31 (40.2%)	29 (37.7%)
	Mandible	52 (42.3%)	11 (8.9%)	13 (10.6%)	30 (24.4%)	17 (13.8%)

**Table 4:** Number of implant insertions and losses within the underlying diseases.

		Malignancy (*n* = 113)	Benign Tumour (*n* = 10)	Malformation (*n* = 24)	Osteonecrosis (*n* = 12)	Others (*n* = 41)
Insertions	None	79 (69.9%)	0 (0.0%)	1 (4.2%)	6 (50.0%)	2 (4.9%)
	1 implant	1	0	1	0	3
	2 implants	5	3	6	1	3
	3 implants	7	4	3	2	8
	4 implants	13	2	8	2	16
	5 implants	5	1	1	1	4
	6 implants	2	0	3	0	4
	7 implants	0	0	0	0	0
	8 implants	1	0	1	0	1
Losses	None	24/34 (70.6%)	9/10 (90.0%)	15/23 (65.2%)	3/6 (50.0%)	33/39 (84.6%)
	1 implant	4	1	4	2	4
	2 implants	2	0	1	1	1
	3 implants	2	0	3	0	0
	4 implants	1	0	0	0	1
	5 implants	1	0	0	0	0

**Table 5:** Complications and reoperations.

	Complication with Osteosynthesis Material	Soft Tissue Correction	Complication with Bone Flap	Bone Flap Loss	Tumour Recurrence (Site of Origin)	Other	Tumour Recurrence (Outside Reconstruction Area)	Other (Next to Reconstruction Area)	New/Additional Bone Flap
Malignancy	17 (15.0)	36 (31.9)	9 (8.0)	8 (7.1)	10 (8.8)	7 (6.2)	6 (5.3)	3 (2.7)	8 (7.1)
Benign tumour	1 (10.0)	4 (40.0)	2 (20.0)	0 (0.0)	-	1 (10)	-	0 (0.0)	0 (0)
Malformation	1 (4.2)	20 (83.3)	1 (4.2)	1 (4.2)	-	0 (0.0)	-	0 (0.0)	2 (8.3)
Osteonecrosis	5 (41.7)	3 (25.0)	0 (0.0)	3 (25.0)	3 (25.0)	1 (8.3)	0 (0.0)	1 (8.3)	4 (33.3)
Other benign disease	5 (12.2)	26 (63.4)	1 (2.4)	0 (0.0)	-	4 (9.8)	-	0 (0.0)	0 (0.0)
*p*-value	0.0713	<0.0001	0.2679	0.0385	0.0163	0.4274	0.5605	0.4423	0.0079
Maxilla	3 (3.9)	47 (61.0)	3 (3.9)	5 (6.5)	4 (5.2)	1 (1.3)	4 (5.2)	1 (1.3)	4 (5.2)
Mandible	26 (21.1)	42 (34.1)	10 (8.1)	7 (5.7)	9 (7.3)	12 (9.8)	2 (1.6)	3 (2.4)	10 (8.1)
*p*-value	0.0007	0.0002	0.3774	1.000	0.7696	0.0183	0.2071	1.000	0.5727
Male	20 (17.5)	50 (43.9)	9 (7.9)	8 (7.0)	7 (6.1)	8 (7.0)	2 (1.8)	3 (2.6)	10 (8.8)
Female	9 (10.5)	39 (45.3)	4 (4.7)	4 (4.7)	6 (7.0)	5 (5.8)	4 (4.7)	1 (1.2)	4 (4.7)
*p*-value	0.2232	0.8861	0.4017	0.5601	1.000	0.7810	0.4054	0.6361	0.4020
No soft tissue paddle	20 (14.0)	70 (49.0)	12 (8.4)	10 (7.0)	6 (4.2)	9 (6.3)	4 (2.8)	2 (1.4)	11 (7.7)
Soft tissue paddle	9 (15.8)	19 (33.3)	1 (1.8)	2 (3.5)	7 (12.3)	4 (7.0)	2 (3.5)	2 (3.5)	3 (5.3)
	0.8244	0.0583	0.1147	0.5149	0.0534	1.000	1.000	0.3213	0.7609

**Table 6:** Correlations of flap sizes and incidence of complications.

	Complication with Osteosynthesis Material	Soft Tissue Correction	Complication with Bone Flap	Bone Flap Loss	Complication with Soft Tissue Paddle	Tumour Recurrence (Site of Origin)	Other	Tumour Recurrence (Outside Reconstruction Area)	Other (Next to Reconstruction Area)	New/Additional Bone Flap
**Rel.Eff.** **(95% CI)**	0.66 (0.55–0.76)	0.36 (0.28–0.44)	0.59 (0.39–0.76)	0.59 (0.43–0.72)	0.62 (0.44–0.77)	0.59 (0.45–0.71)	0.70 (0.53–0.83)	0.50 (0.33–0.67)	0.81 (0.60–0.92)	0.61 (0.47–0.73)
***p*** **-value**	0.0071	0.0009	0.3633	0.2647	0.1870	0.2236	0.0250	0.9963	0.0059	0.1275

## Data Availability

The data presented in this study are available on request from the corresponding author.
